# Simulation, Design, and Test of a Dual-Differential D-Dot Overvoltage Sensor Based on the Field-Circuit Coupling Method

**DOI:** 10.3390/s19153413

**Published:** 2019-08-03

**Authors:** Pengcheng Zhao, Jingang Wang, Qian Wang, Qianbo Xiao, Ruiqiang Zhang, Shucheng Ou, Yaqin Tao

**Affiliations:** 1 State Key Laboratory of Power Transmission Equipment and System Security, Chongqing University, Chongqing 400044, China; 2 Chongqing Electric Power Research Institute, Chongqing Electric Power Company, Chongqing 401123, China; 3 Baotou Power Supply Bureau of Inner Mongolia Electric Power Group Co., Ltd, Baotou 014030, China; 4 State Grid Chongqing Jiangbei Power Supply Company, Chongqing Electric Power Company, Chongqing, 401147, China

**Keywords:** Dual-Differential, D-dot, Field-Circuit Coupling, Overvoltage Measurement

## Abstract

Accurate measurement of overvoltage in power grids is of great significance to study the characteristics of overvoltage and design of insulation coordination. Based on the research of D-dot voltage sensor, we designed a Dual-Differential D-dot overvoltage sensor. In order to quantify the structural parameters of the sensor, improve the performance and measurement accuracy of the sensor. The Field-Circuit Coupling method was proposed to be used in the parameter design of D-dot overvoltage sensor. The joint simulation of space electromagnetic field model and equivalent circuit model of the Dual-Differential D-dot overvoltage sensor was established with the finite element simulation software Ansoft Maxwell and circuit simulation software Simplorer. Finally, the actual sensor was manufactured. A test platform was built to verify the steady-state and transient performance of the sensor. The results show that the Dual-Differential D-dot sensor has excellent steady-state and transient performance, the error of phase and amplitude are small, and the sensor can achieve the non-contact measurement of power transmission line. Simultaneously, the rationality of the Field-Circuit Coupling method was further verified.

## 1. Introduction

Overvoltage is an important factor threatening the safe and stable operation of substations and transmission lines. Overvoltage in a high-voltage power grid is difficult to be measured due to its large amplitude and high frequency [1,2,3]. Scholars at home and abroad have proposed many calculation models and measurement methods for overvoltage measurement and protection [4,5,6,7], but there is a lack of overvoltage waveform and parameters. Therefore, the study of an overvoltage sensor with convenient installation, fast response, wide measurement bandwidth, strong anti-interference capability to sense the overvoltage waveform and parameters safely and accurately, which provides references for analyzing substation overvoltage accident, improving overvoltage prevention measures, enhancing the insulation coordination of overhead transmission power line. The study is of great significance for ensuring the safe operation of power system [8,9].

In the existing overvoltage measurement methods, the resistance divider has the problem of thermal capacity, and the measurement error is large. It is only suitable for voltage with low frequency and amplitude. Using capacitive voltage divider or resistance-capacitance voltage divider to detect bus overvoltage, although the divider has the advantages of high measurement accuracy and good transient response characteristics, the long-term parallel connection of the divider to bus operation will bring many potential risks to the high-voltage power grid [10,11,12]. The new type of optical fiber sensor exists inevitable drawbacks in reliability, long-term operation stability, and environmental temperature impact, and it also needs to be connected to primary equipment and there is a non-linear problem in photoelectric conversion [13,14,15,16]. The D-dot sensor is only suitable for measuring overvoltage signal at specific high frequencies and the measurement bandwidth is small, the improved differential D-dot sensor is mostly used for steady-state measurement, and cannot capture fast high-frequency transient signal [17,18]. Therefore, it is of great practical significance to develop suitable sensors for accurate measurement of overvoltage.

Based on the analysis of the existing overvoltage measurement methods, a Dual-Difference D-dot overvoltage sensor based on the electric Field-Coupling method is proposed. We designed the sensor composed of four electrodes. The Field-Circuit coupling method is applied to the sensor parameter design to determine the optimal parameters of the sensor. It solves the problem of blindness, consistency, and unquantifiable parameters in the sensor design process. Finally, based on the sensor parameters obtained from the simulation analysis, we made the actual sensor. In order to verify the performance of the sensor, a test platform was built with overhead transmission lines as the measurement object. The results indicate that the sensor has the characteristics of fast response and small phase and amplitude error. Both steady state voltage signal and transient overvoltage signal can be measured accurately. At the same time, it also proves the rationality of the Field-Circuit coupling method to guide the sensor design. This method can not only be used in the design of D-dot overvoltage sensor, but also can be used for reference in the design of other types of sensors.

## 2. Principle of the Dual-Differential D-Dot Overvoltage Sensor

### 2.1. Measurement Principle of the Sensor

**Figure 1:**
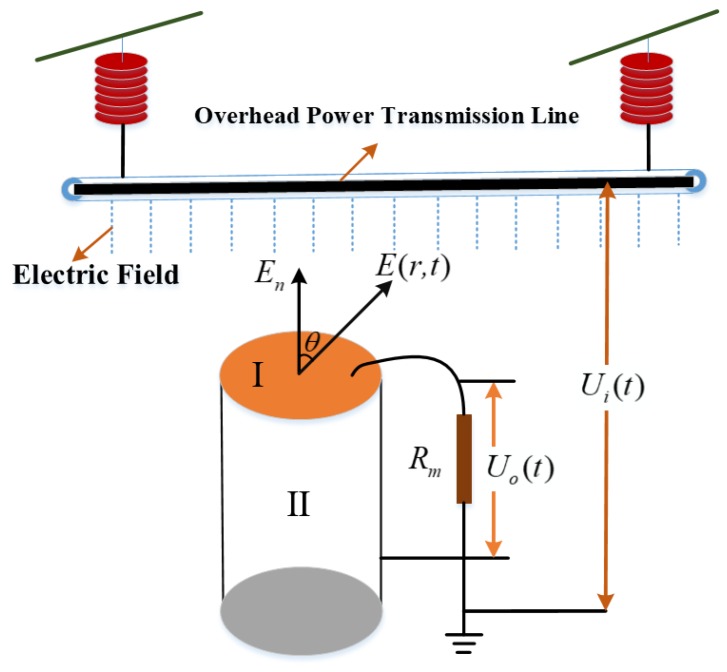
Measurement principle of traditional D-dot voltage sensors.

For a traditional monopole D-dot voltage sensor with any shape can be approximately equivalent to the structure shown in Figure 1. The measurement of the voltage signal can be achieved by measuring the change rate of the electrical displacement vector.

In Figure 1, the sensor is put in a space with the electric field intensity of *E*(*r, t*). I is a metal electrode of any shape with an external surface of A. II is a metal insulator with metal electrode mounted on it. The metal electrode is connected with the grounded equivalent measuring resistance *R_m_* through coaxial cable [19,20]. The relationship between the single electrode output *U_o_*(*t*) and the measured wire potential *U_i_*(*t*) can be deduced from the Gauss theorem [21]. The output voltage of the sensor can be expressed as:

(1) $$U_{o}(t)=R_{m}A_{eq}\frac{dU_{i}(t)}{dt}$$

In Equation (1), *A_eq_* is the equivalent area of the sensor measuring electrode, *U_i_*(*t*) is the transmission line voltage to be measured, and *U_o_*(*t*) is the output voltage signal of the sensor. The voltage of the transmission line voltage can be obtained by integrating the output signal of the sensor. Thus, the non-contact measurement of transmission line voltage can be realized.

**Figure 2:**
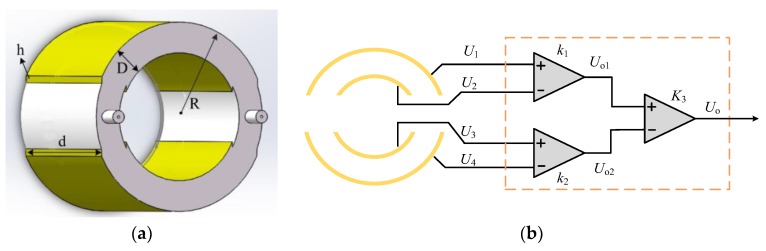
Parameter structure and measurement principle of the Dual-Differential D-dot overvoltage sensor: (**a**) The structural parameter of the Dual-Differential sensor; (**b**) the signal measurement principle of the sensor.

In order to improve the sensor's ability to capture the surrounding electric field and avoid the interference caused by the introduction of grounding resistance [22,23,24,25,26], a Dual-Differential D-dot overvoltage sensor is designed. Figure 2 is the parameter and measurement principle of the Dual-Differential D-dot overvoltage sensor.

In Figure 2a, R is the outer radius of the sensor, D is the distance between electrodes, d is the width of the electrode, and h is the thickness of the electrode. In Figure 2b, the power transmission line voltage *U_o_*(t) can be obtained by differential amplification of each electrode signal. *U_o1_(t)*, *U_o2_(t),* and *U_o_*(t) can be expressed as:

(2) $$U_{o1}(t)=k_{1}[U_{1}(t)-U_{2}(t)]=k_{1}R_{m1}(A_{eq1}-A_{eq2})\frac{dU_{i}(t)}{dt}$$

(3) $$U_{o2}(t)=k_{2}[U_{3}(t)-U_{4}(t)]=k_{2}R_{m2}(A_{eq3}-A_{eq4})\frac{dU_{i}(t)}{dt}$$

The *U_o_*(t) can be obtained from Equations (2) and (3):

(4) $$U_{o}(t)=k_{3}[U_{o1}(t)-U_{o2}(t)]=k_{3}\frac{dU_{i}(t)}{dt}[k_{1}R_{m1}(A_{eq1}-A_{eq2})-k_{2}R_{m2}(A_{eq3}-A_{eq4})]$$

Equation (4) is derived from the equivalent circuit in Figure 2b, and the differential amplification module in Figure 2 is the INA111 instrumentation amplifier from Texas Instruments (TI). The INA111 is a high speed, FET(Field Effect Transistor)-input instrumentation amplifier offering excellent performance. It can suppress the common mode signal and amplify the differential input signal. At the same time, because of the Dual-Differential structure of four plates, the sensor has a larger contact area with the surrounding electric field. According to Equations (1) and (4), the larger equivalent contact area *A_eq_* means better voltage amplification capability; therefore, the sensor can capture more voltage signals even in weak electric field environment, having stronger adaptability. Compared with the monopole D-dot voltage sensor, the Dual-Differential D-dot overvoltage sensor is not connected to a grounding resistance, but a resistor is connected between the electrodes. It can avoid the influence of the grounding resistance.

### 2.2. Equivalent Circuit of Sensor

**Figure 3:**
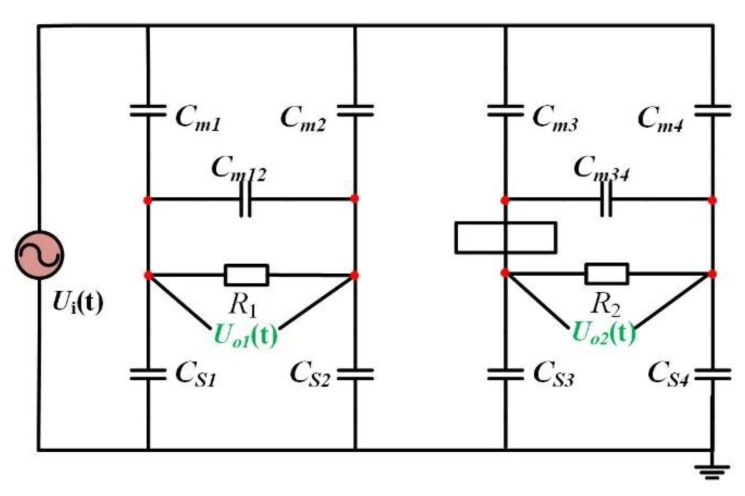
Equivalent circuit of the Dual-Differential D-dot overvoltage sensor.

The equivalent circuit of the sensor when measuring the voltage of transmission line is shown in Figure 3.

In Figure 3, *U_i_*(t) is the voltage of the transmission line to be measured, nodes 1, 2, 3, and 4 are the four electrode measuring points of the sensor. *C_m1_*, *C_m2_*, *C_m3_*, and *C_m4_* are the mutual-capacitance between the four electrodes and the line to be measured, *C_s1_*, *C_s2_*, *C_s3,_* and *C_s4_* are the stray-capacitance from the four electrodes to the ground, and *C_m12_* and *C_m34_* are the mutual capacitance between the two pairs of electrodes. *R_m1_* and *R_m2_* are the input equivalent resistances of the differential amplifiers. From this equivalent circuit, it can be deduced that the transfer function of the Dual-Differential D-dot overvoltage sensor is as follows:

(5) $$H(s)=\frac{U_{o}(s)}{U_{i}(s)}=\frac{s[sR_{1}R_{2}(C_{1}C_{4}-C_{2}C_{3})+(R_{1}C_{1}-R_{2}C_{3})]}{\left(sR_{1}C_{2}+1)(sR_{2}C_{4}+1\right)}$$

where:

(6) $$C_{1}=\frac{C_{m1}C_{s2}-C_{m2}C_{s1}}{C_{m1}+C_{m2}+C_{s1}+C_{s2}}.$$

(7) $$C_{2}=\frac{1}{\frac{1}{C_{m1}+C_{s1}}+\frac{1}{C_{m2}+C_{s2}}}+C_{m12}$$

(8) $$C_{3}=\frac{C_{m3}C_{s4}-C_{m4}C_{s3}}{C_{m3}+C_{m4}+C_{s3}+C_{s4}}.$$

(9) $$C_{4}=\frac{1}{\frac{1}{C_{m3}+C_{s3}}+\frac{1}{C_{m4}+C_{s4}}}+C_{m34}$$

This transfer function is used to calculate the input and output amplitude-frequency response curves of the sensor to determine the measurement bandwidth of the sensor. 

## 3. Design Method and Implementation of Field-Circuit Coupling

The Field-Circuit Coupling method (solving the equivalent measurement circuit equation of the sensor and the electric field coupling equation around the transmission line) is used to determine the optimal parameters of the sensor. The following is the specific process: Firstly, building a single-phase transmission line model through the finite element analysis software Ansoft Maxwell. The voltage excitation is applied to the power transmission line to obtain an electric field distribution around it. Secondly, the sensor model is introduced into the electric field environment formed by the transmission line to calculate the output voltage value. Then the simulation results are substituted into the sensor equivalent measurement circuit. Verifying the sensor's performance is verified by solving the sensor's transfer function. Finally, determine the optimal parameters of the sensor through continuous iterative calculation.

### 3.1. Field-Circuit Coupling Method

**Figure 4:**
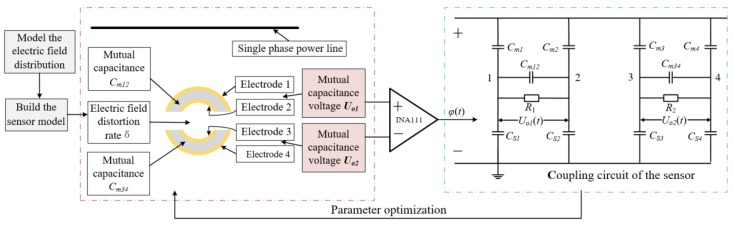
Implementation steps of Field-Circuit Coupling method.

The implementation steps of the Field-Circuit Coupling method are shown in Figure 4.

A single-phase power transmission line model is established in the limit element analysis software Ansoft Maxwell, and a steady-state voltage excitation and a transient overvoltage surge signal are applied to the power transmission line respectively, the electric field distribution around the power transmission line is obtained. The Dual-Differential D-dot overvoltage sensor was modeled in Ansoft Maxwell, and the sensor model was placed in the electric field environment formed by the transmission line for simulation calculation. Then, the equivalent circuit of the sensor is established in the software Simplorer. Then the transfer function is analyzed and calculated. By analyzing the simulation results, if the error is within the allowable range, it is proved that the designed sensor parameters meet the requirements. However, if the error is large, the parameters of the sensor model need to be further adjusted. Finally, the structural parameters, such as sensor electrode size and the distance between different electrode, are determined at the minimum error to achieve a more accurate measurement of the Dual-Differential D-dot overvoltage sensor.

### 3.2. Joint Simulation Calculation

**Figure 5:**
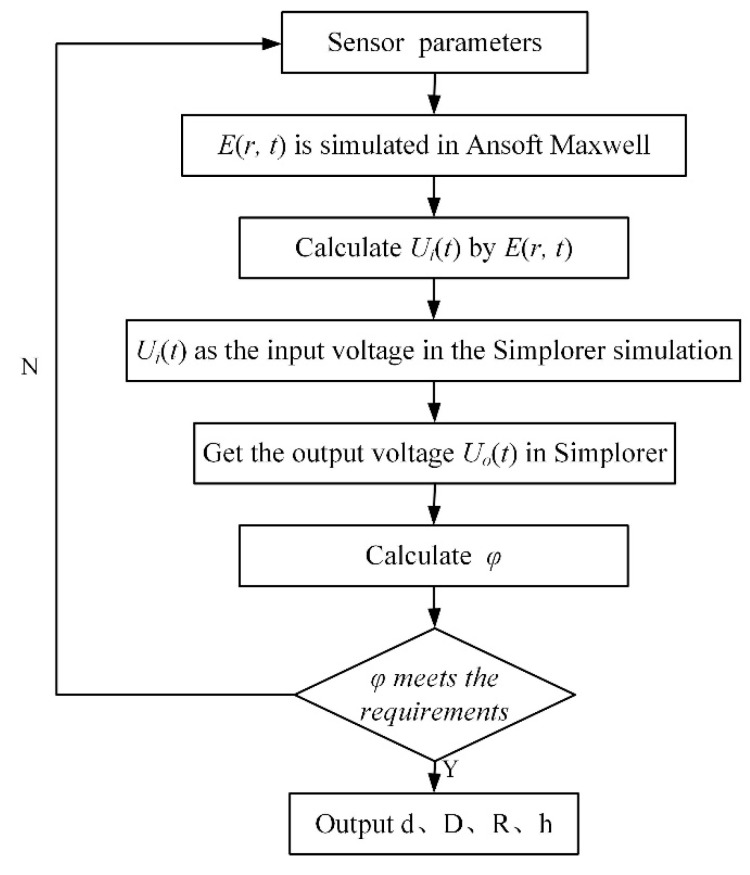
Joint simulation flow chart of the Field-Circuit analysis method.

The key to optimizing the parameters of the Dual-Differential D-dot overvoltage sensor is to establish the corresponding models by finite element simulation software Ansoft Maxwell and circuit simulation software Simplorer. Taking the phase difference between the output voltage and the input voltage in the Simplorer is taken as the optimization target (the phase difference φ shall be less than 1°). Determine the capacitance value based on the sensor parameters, and the relevant capacitance parameters of the sensor in the equivalent circuit were determined, and the structural parameters of the sensor model in Ansoft Maxwell were adjusted based on the above data. Then, the parameters were simulated to verify whether the measurement requirements are met, and the suggestions for parameter modification were fed back to the circuit simulation software Simplorer. Through the continuous transmission and feedback of data between the two simulation models, the parameters and measurement performance of the sensor were improved and optimized. The joint simulation flow chart using field-circuit analysis method is shown in Figure 5.

**Figure 6:**
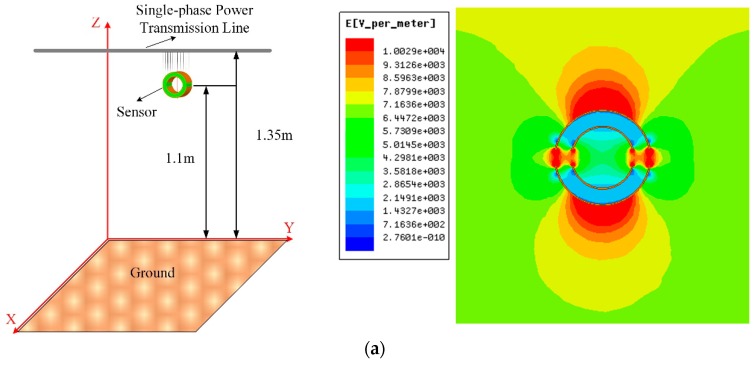

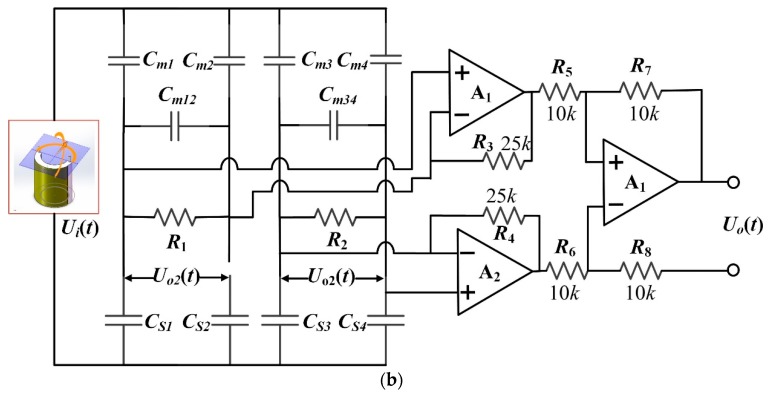
Physical model of Field-Circuit coupling and circuit connection diagram: (**a**) Sensor simulation model and electric field distribution; (**b**) Equivalent circuit connection diagram.

The simulation diagram of the Dual-Differential D-dot overvoltage sensor in Ansoft Maxwell and the circuit connection diagram in Simplorer are shown in Figure 6. Figure 6a is the simulation model of sensor in Ansoft Maxwell, and Figure 6b is the simulation circuit diagram of sensor equivalent circuit in Simpler. The voltage excitations of different levels (1 kV to12 kV) were applied to the power transmission line in Figure 6a, and the electric field distribution was projected into the yoz plane. The electric field strength around the sensor electrode is about 6945 C/m^2^, the electric field strength in the same area is about 7123 C/m^2^ when the sensor is not placed in the electric field. Electric field distortion rate is 2.499%, therefore, the introduction of the sensor has less influence on the electric field distortion. At the same time, it can be seen from the data in the Figure 6b that the highest internal electric field strength is 10.029 kV/m. The material filled between the electrodes is epoxy resin, and the breakdown strength of the epoxy resin is 35 MV/m. So, the insulation performance of the sensor meets the demand.

**Figure 7:**
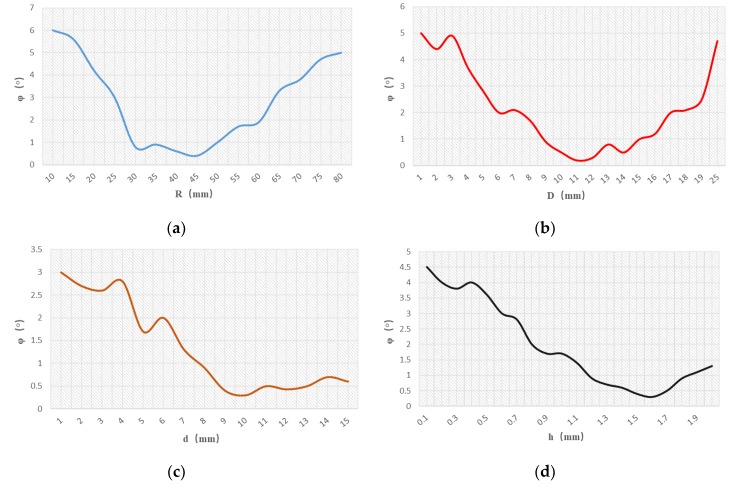
Characteristic curves of sensor parameters and phase difference: (**a**) R-φ. (**b**) D-φ. (**c**) d-φ. (**d**) h-φ.

In the process of simulation, the parameters of the outer radius R, distance between electrodes D, electrode width d, and electrode thickness h of the sensor were changed to obtain the electric field value around the sensor. The output voltage of the sensor can be obtained by partial differentiation of the electric field value and multiplication by the corresponding proportional coefficient. Simultaneously, the phase difference φ between the input voltage and the output voltage of the equivalent circuit was calculated by simulation in Simpler. The characteristic curve of the phase difference between the sensor parameters and the phase difference φ was obtained by iterative calculation as shown in Figure 7.

As can be seen from Figure 7 that the outer radius R is between 30 and 45 mm, the distance between electrodes D is between 4 and 12 mm, the electrode width d is between 50 and 80 mm, the electrode thickness h is between 1.5 and 1.8 mm, the phase difference of the Dual-Differential D-dot sensor has minimum, which meets the design requirements of the sensor.

### 3.3. Parameter Determination and Production of Sensor

The smaller distance between electrodes and wider width can increase the parameter values of mutual capacitance between electrodes, improving the performance of the sensor. Combined with the relevant characteristic curves in Figure 7, it can be found that when the width of the electrode reaches 50 mm, the output phase difference of the sensor is small. However, considering that the sensors should be easy to install in complex environments during practical application, the parameter d was selected as 55 mm in the design. At the same time, in order to ensure the durability of the sensor, the parameter D was selected as 11 mm. In summary, the structural parameters of the sensor were determined as shown in Table 1.

**Figure 8:**
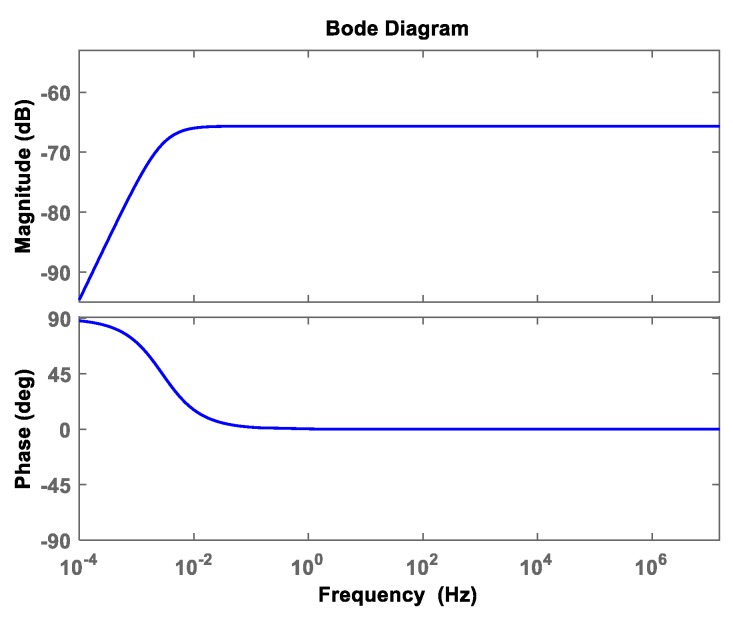
Response curves of amplitude-frequency and phase-frequency of the sensor.

**Figure 9:**
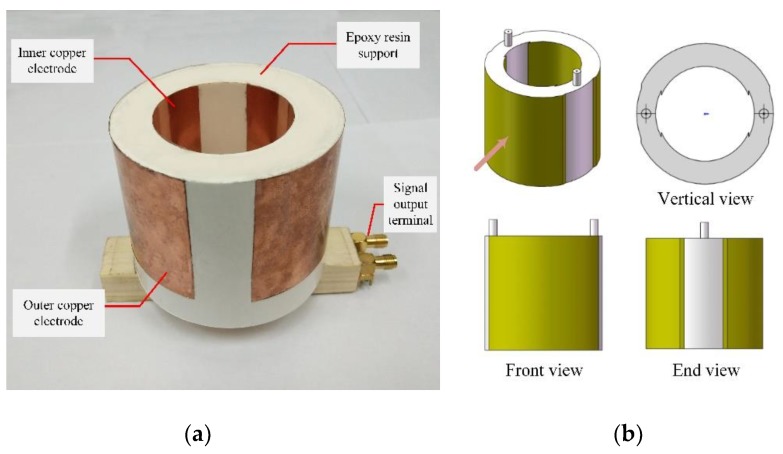
The physical picture and orthographic views of Dual-Differential D-dot sensor: (**a**) Physical picture; and (**b**) Orthographic views.

The capacitance value of the sensor can be calculated according to the parameters of the sensor in Table 1. The results are shown in Table 2:

The amplitude and phase response curves of the sensor can be obtained by substituting the parameters in Table 2 into Equation (5). The response curve is shown in Figure 8. As can be seen from Figure 8, the sensor has a stable gain of MHz level and the phase difference is close to 0°. The designed sensor can measure broadband overvoltage signals, which verifies the rationality of sensor parameter design. Finally, according to the parameters in Table 1, the sensor model was made as shown in Figure 9. The supporting material in Figure 9 is epoxy resin. The manufacturer is Huipin, item number HL-807-1. The maximum electric field strength that epoxy resin can withstand is 35 MV/m, which has good dielectric strength and can meet the actual measurement demand.

## 4. Experiment Verification

After the design of the Dual-Differential D-dot overvoltage sensor is ended, in order to verify the performance of the sensor, a test platform was built and the following tests were completed: (1) Steady-state performance test; (2) Overvoltage impulse transient test.

### 4.1. Test Method

**Figure 10:**
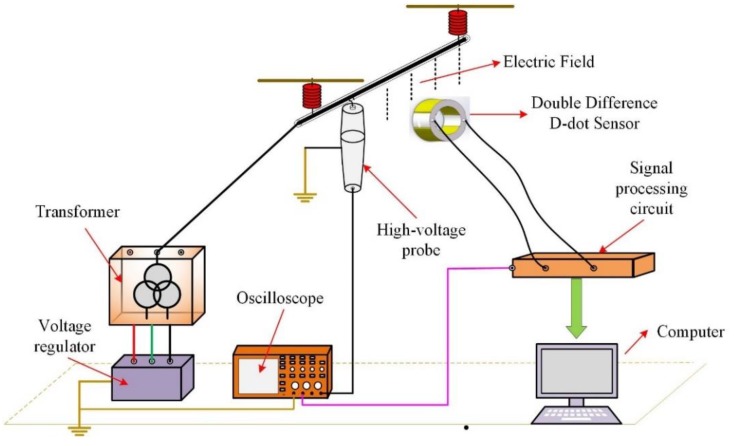
The structure diagram of the test platform.

**Figure 11:**
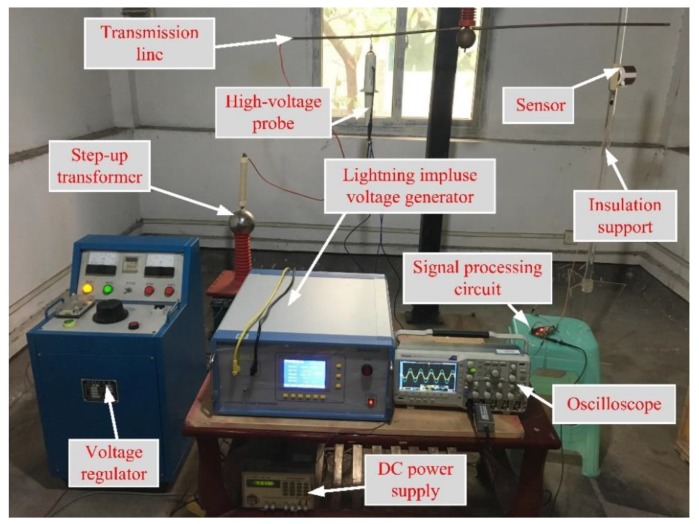
The sensor test platform.

The structure diagram of the test platform and physical picture are shown in Figure 10 and Figure 11. The Dual-Differential D-dot overvoltage sensor was placed under the power transmission line, with output terminal of the sensor connected to the signal processing circuit, the signal processing circuit connected to the oscilloscope to measure the real-time waveform. Changing the power transmission line voltage value through the transformer console. In the steady-state test, the console outputs a voltage of 50 Hz to the power transmission line, and the voltage value was measured by the Dual-Differential D-dot overvoltage sensor to verify its steady-state performance. In the transient test, the 10-kV lightning impulse voltage generator (SUG255X) was used as the standard lighting overvoltage source to verify the transient characteristics of the sensor. The high-voltage probe of Tektronix P6015A was selected for sensor calibration.

### 4.2. Steady-State Performance Tests

**Figure 12:**
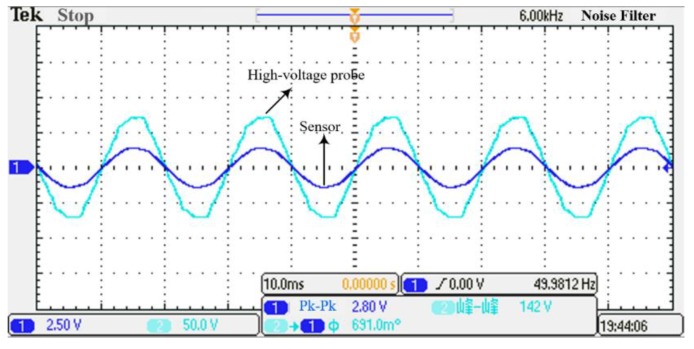
Steady-state measurement waveform at 5 kV.

The voltage applied on the power transmission line was successively increased by the test platform, and the effective voltage measurement range was 1 kV to 12 kV. The voltage signal measured by the sensor was input to channel 1 of the oscilloscope, and the power transmission voltage measured by the high-voltage probe was input to channel 2 of the oscilloscope. Taking the phase voltage waveform with an effective value of 5 kV as an example, the waveform comparison measured by the high-voltage probe and the sensor is as shown in Figure 12.

From Figure 12, it can be seen that the voltage waveform measured by the designed the Dual-Differential D-dot overvoltage sensor is basically the same as the output waveform of the high-voltage probe. The waveform distortion and the phase difference of the sensor is small, which indicates that the sensor has good steady-state characteristics. The distortion of the waveform, measured by the high-voltage probe, is caused by interference from the surrounding measurement environment and the unstable power quality. The Dual-Differential D-dot sensor adopts a Dual-Differential structure, which can amplify the differential mode signal while suppressing the common mode signal. The sensor will be less disturbed by the surrounding environment in this way. When the sensor is carried out in steady-state experiments, the filter circuit we used has a narrow bandwidth, and the cut-off frequency is 55 Hz. As a result, these disturbance signals are filtered out, and no disturbance occurs to the waveform measured by the sensor.

The ratio error and phase difference of the sensor were measured and calculated at voltage levels of 1 kV, 2 kV, 3 kV, 5 kV, 6 kV, 9 kV, and 10 kV, respectively. These results are shown in Table 3. Where, the ratio error $\varepsilon \%=\frac{K_{n}U_{S}-U_{H}}{U_{H}}\times 100\%$, *U_H_* and *U_S_* are the test data measured by the high-voltage probe and the Dual-Differential D-dot overvoltage sensor, respectively.

Fitting the data of *U_H_* and *U_S_* in Table 3, the linear correlation coefficient is close to 1, which indicates that the designed sensor has excellent linearity. At the same time, we can see that the sensor's ratio error is less than 0.8%, which indicates that the distortion of the measured waveform of the sensor is extremely small and has satisfactory steady state performance.

### 4.3. Overvoltage Impulse Transient Tests

**Figure 13:**
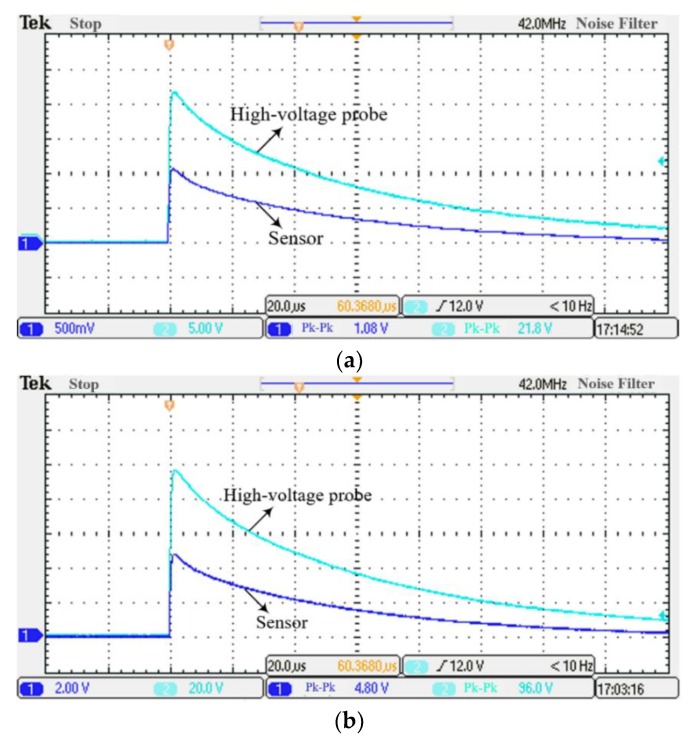
Transient measurement waveform of the Dual-Differential D-dot overvoltage sensor; (**a**) 2 kV, (**b**) 9 kV.

In order to test the transient performance of the sensor, a surge voltage of 2 kV to 10 kV is applied to the power transmission line. In the test, the lightning impulse wave was simultaneously tested by the Dual-Differential D-dot overvoltage sensor and the high-voltage probe. When the peak voltage of the lightning wave is taken as 2 kV and 9 kV, the waveform is shown in Figure 13.

It can be seen from Figure 13, that when the peak voltage of lightning wave is 2 kV, the peak response time of high-voltage probe and sensor is 1.21 *us* and 1.23 *us*, respectively. When the peak voltage is 9 kV, the peak response time of the high-voltage probe and sensor is 1.21 *us* and 1.22 *us*, respectively. The relative errors of the time at which the sensor responds to the peak are both less than 1.7%. By comparing the transient output waveforms of the high-voltage probe and the sensor, we can find that the peak voltage response time of the two is basically same. The waveform trend of the sensor and high-voltage probe is consistent. This indicates that the sensor has a fast response speed and no high frequency oscillation.

The results of transient response test at 2 kV–10 kV are shown in Table 4. *U_H-PK_(CH1)* and *U_S-PK_(CH2)* are the peak values of lightning waves measured by the sensor and high-voltage probe, respectively.

When a standard lightning impulse voltage of 2 kV to 10 kV is applied, the relative peak error of the output voltage between the sensor and the high-voltage probe is less than 1.9%. It shows that the sensor can achieve high-precision measurement of overvoltage signals. The test results indicate that the Dual-Differential D-dot overvoltage sensor has good transient measurement performance and can effectively measure the overvoltage of the power transmission line.

## 5. Conclusion

Based on the research of D-dot voltage sensor, we designed a Dual-Differential D-dot overvoltage sensor. The Field-Circuit Coupling method was used to optimize the parameters of the sensor. The joint simulation of the sensor equivalent circuit model and the electric field coupling model around the transmission line was established. Finally, the optimal parameters of the sensor were determined and the actual sensor was made.

In order to verify the performance of the sensor, a steady-state performance test and overvoltage impulse transient test were carried out. The steady-state test shows that the Dual-Differential D-dot overvoltage sensor can measure the steady-state signals quickly and accurately, comparing with the high voltage probe, the phase error is less than 0.7° and the ratio error is less than 0.8%. The transient test shows that the sensor has good transient response capability. Comparing the peak signal measured by the high-voltage probe, the relative error of the response time is less than 1.6%, and the peak error of the sensor is less than ±1.9%, which indicates that the sensor can accurately measure the overvoltage signal. At the same time, the reasonability of the Field-Circuit Coupling method has been proved. The proposed design concept of applying the Field-Circuit Coupling method to the Dual-Differential D-dot overvoltage sensor also provides guidance to the parameter design of other types of sensors.

## Figures and Tables

**Table 1:** Structural parameters of the Dual-Differential D-dot sensor.

	R	h	D	d
**Outer electrode**	45	1.6	11	55

**Table 2:** The capacitance value of the Dual-Differential D-dot sensor.

C*_m1_*/*pF*	C*_m2_*/*pF*	C*_m3_*/*pF*	C*_m4_*/*pF*	C*_s1_*/*pF*
2.5617	2.2731	2.3126	2.4083	1.7114
**C*_s2_*/*pF***	**C*_s3_*/*pF***	**C*_s4_*/*pF***	**C*_m12_*/*pF***	**C*_m34_*/*pF***
1.6852	1.6221	1.8054	15.253	16.091

**Table 3:** Test results of steady-state response.

Voltage/kV	*U*_H_/kV	*U*_S_/V	ε% (±)	*φ*/(°)
**1**	1.012	0.21	0.79	0.1699
**2**	2.034	0.42	0.59	0.1726
**3**	2.998	0.62	−0.73	0.5187
**5**	5.020	1.05	0.40	0.6910
**6**	5.798	1.21	0.45	0.3455
**9**	8.555	1.78	−0.13	0.5117
**10**	9.899	2.06	0.13	0.3455

**Table 4:** Test results of transient response.

*U_H-PK_/kV*	*U_S-PK_*/V	Relative Error/%
2.16	1.10	1.85
3.28	1.62	1.22
4.32	2.19	1.81
5.44	2.68	1.47
6.56	3.29	0.30
7.52	3.82	1.59
8.56	4.32	0.93
9.60	4.80	0.20
10.70	5.36	0.19
